# Maternal Depressive Symptoms Not Associated with Reduced Height in Young Children in a US Prospective Cohort Study

**DOI:** 10.1371/journal.pone.0013656

**Published:** 2010-10-27

**Authors:** Karen A. Ertel, Karestan C. Koenen, Janet W. Rich-Edwards, Matthew W. Gillman

**Affiliations:** 1 Department of Society, Human Development, and Health & Kellogg Health Scholars Program, Harvard School of Public Health, Boston, Massachusetts, United States of America; 2 Department of Epidemiology, Harvard School of Public Health, Boston, Massachusetts, United States of America; 3 Center on the Developing Child, Harvard University, Cambridge, Massachusetts, United States of America; 4 Center for Women's Health and Gender Biology, Brigham and Women's Hospital, Boston, Massachusetts, United States of America; 5 Obesity Prevention Program, Department of Population Medicine, Harvard Medical School and Harvard Pilgrim Health Care Institute, Boston, Massachusetts, United States of America; 6 Department of Nutrition, Harvard School of Public Health, Boston, Massachusetts, United States of America; University of Cape Town, South Africa

## Abstract

**Background:**

Shorter stature is associated with greater all cause and heart disease mortality, but taller stature with increased risk of cancer mortality. Though childhood environment is important in determining height, limited data address how maternal depression affects linear growth in children. We examined the relationships between antenatal and postpartum depressive symptoms and child height and linear growth from birth to age 3 years in a U.S. sample.

**Methods:**

Subjects were 872 mother-child pairs in Project Viva, a prospective pre-birth cohort study. The study population is relatively advantaged with high levels of income and education and low risk of food insecurity. We assessed maternal depression at mid-pregnancy (mean 28 weeks' gestation) and 6 months postpartum with the Edinburgh Postnatal Depression Scale (score > = 13 on 0–30 scale indicating probable depression). Child outcomes at age 3 were height-for-age z-score (HAZ) and leg length. HAZ was also available at birth and ages 6 months, 1, 2, and 3 years.

**Findings:**

Seventy (8.0%) women experienced antenatal depression and 64 (7.3%) experienced postpartum depression. The mean (SD) height for children age 3 was 97.2 cm (4.2), with leg length of 41.6 cm (2.6). In multivariable linear regression models, exposure to postpartum depression was associated with greater HAZ (0.37 [95% confidence interval: 0.16, 0.58]) and longer leg length (0.88 cm [0.35, 1.41]). The relationship between postpartum depression and greater HAZ was evident starting at 6 months and continued to age 3. We found minimal relationships between antenatal depression and child height outcomes.

**Conclusion:**

Our findings do not support the hypothesis that maternal depression is associated with reduced height in children in this relatively advantaged sample in a high-income country.

## Introduction

Extant literature largely from the United States and Western European nations reveals that body height is strongly associated with health outcomes. Shorter stature is associated with increased risk of mortality,[1]–[5] as well as mortality due to cardiovascular[1], [2] and respiratory disease.[1], [4] At the other end of the spectrum, tall individuals have greater risk for cancer and mortality due to cancer.[4], [6]–[9] The underlying reasons for these associations are unknown, but two possible mechanisms are that adult height reflects growth, nutrition, and the social environment in early life and that height in adulthood is positively associated with social class and its attendant exposures.[1] Leg length, a component of height, may be a more sensitive marker of prepubertal environmental influences than total height because height increases in childhood are due more to leg growth than trunk growth.[10] Similar to total height, leg length is inversely associated with cardiovascular risk factors and coronary heart disease,[6], [7], [11]–[13] but positively associated with cancer risk.[6]

Height in childhood is strongly correlated with adult height: at age 2 years, length or height has a correlation of 0.8 with adult height.[14] Similar to attained height in adulthood, accelerated linear growth in childhood has been positively associated with breast cancer risk.[15], [16] Additionally, a recent analysis of over 3,000 young girls in the United States revealed that greater height in childhood (ages 4–5 years) predicted early menarche,[17] which is linked to adverse mental and physical health outcomes.[15], [18], [19]

The current literature regarding determinants of height focuses on developing countries. This is critically important, as nutritional status, disease, and infrastructure have significant influences on attained height in this context. However, the evidence reviewed above for the associations between height and adult health outcomes comes largely from the United States or Western European nations, thus we believe there is value in continued investigation of determinants of linear growth in the context of developed countries, such as the United States. Though many factors contribute to final height, including genetics, nutrition, and disease,[20] less attention has been paid to potential social and psychosocial influences on linear growth. Maternal mental health is a primary determinant of the child's psychosocial environment. Maternal depression is common in the postpartum period, with an estimated 14% of new mothers experiencing depression in the first 6 months after delivery.[21] Given its prevalence and its documented effect on child environment,[22], [23] mother-child interaction,[24]–[26] and child physiology,[27]–[29] maternal depression may be an important determinant of linear growth in young children.

Previous studies using underweight or non-organic failure to thrive as indicators of poor physical growth have suggested an association between maternal depression and inadequate growth, but results have not consistently supported this association [30]–[37](for a review of recent findings, see Stewart et al, 2007[38]). The literature specific to height is smaller and largely in developing countries; these studies provide mixed support for an association between postpartum depression and reduced child linear growth. Studies in India,[39] Bangladesh,[40] Brazil,[41] and Nigeria[42] have shown an association between maternal depressive symptoms and child stunting, while studies in South Africa[43] and Brazil[44] have shown no significant difference in child height according to maternal depressive symptoms. Similar methodology was used in four developing countries to examine maternal mental health (not depression per say) and child stunting; they found an independent, cross-sectional association between maternal ‘common mental disorder’ and stunting in India, but not Ethiopia, Peru, or Vietnam.[45] It is difficult to compare across studies due to different measures of maternal depression and ages of assessment of children. Further complicating interpretation is the possibility of reverse causation, as most of these studies were cross-sectional.

Antenatal depression, through its effects on fetal growth, may also influence child stature. Antenatal depression, with a period prevalence of 18% during pregnancy,[21] is associated with low birth weight, preterm birth, and small for gestational age at birth.[46] Infants born small tend to remain small in height as well as weight through childhood,[47] suggesting that an adverse intrauterine environment may contribute to short stature. A prospective cohort study in India reported 3.2 and 2.8 increased odds of stunting at 6 months and 12 months, respectively, for children whose mothers were depressed in the 3^rd^ trimester of pregnancy compared to children whose mothers were not depressed at that time.[48]

Based on this prior literature, we hypothesized that children whose mothers experienced antenatal or postpartum depression would be shorter than children of mothers without depression. This study is among the first to examine these associations in a high-income country. Additionally, the sample population for this study differs from those in which an association has been found between maternal depression and stunting in several important ways, including higher levels of family income, access to sophisticated health services, low prevalence of food insecurity and hunger, low rates of infection, high levels of education, and low rates of both depression and stunting. Thus, this analysis may represent a rather stringent test of the association between maternal depression and child height.

## Methods

### Sample and data collection

Subjects were from Project Viva, a prospective cohort study of pregnant women and their singleton children. Participants were recruited at their first prenatal visit from eight obstetric practices of Harvard Vanguard Medical Associates, a large group practice in the greater Boston area. Exclusion criteria included multiple gestation, inability to answer questions in English, plans to move out of the area before delivery, and gestational age more than 22 weeks at first prenatal visit. 2670 pregnant women (64% of those eligible) were enrolled between April 1999 and July 2002; 329 subsequently became ineligible (60% because they were no longer pregnant). Of the remaining 2341 enrolled women, 195 withdrew and 18 were lost to follow-up before delivery. 2128 participants delivered a live infant and were eligible for inclusion in the current analysis. 1635 (70%) completed a mid-pregnancy questionnaire that assessed depressive symptoms and 1422 (60% of those enrolled) completed a second questionnaire that assessed depressive symptoms at 6 months postpartum. 1249 women (53% of those enrolled) had data on both antenatal and postpartum depression. Data were also collected at birth, including enrollment of the child, and at, 1, 2, and 3 years after delivery. Among participants with depressive symptom data at mid-pregnancy and 6 months postpartum, 928 had height data for children at age 3 years. Of these, we excluded 56 (6.0%) due to missing covariate information for a final sample size of 872. Comparison of the 872 subjects included in this analysis to those excluded revealed some differences: included mothers were more likely to be white (79.6% vs. 74.3%), more highly educated (38.4% vs. 22.1% with a graduate degree), slightly older (mean age 33 years vs. 31 years), and slightly taller (mean height 65.1 vs. 64.7 inches). The Project Viva study and consent procedures were approved by human subjects committees of Harvard Pilgrim Health Care, Brigham and Women's Hospital, and Beth Israel Deaconess Medical Center. Written informed consent was obtained from all mothers in the study.

### Measures

#### Depressive symptoms

We assessed depressive symptoms with the 10-item Edinburgh Postnatal Depression Scale (EPDS) in mid-pregnancy (mean of 28 weeks' gestation) and at approximately 6 months after birth. We chose the EPDS because it has been validated for antenatal and postpartum use.[49] We used the cutpoint of 13 or more (on the 0–30 point scale) to indicate probable depression, consistent with previous work in our cohort [50], [51] and in other large cohorts that collected EPDS data antenatally and postnatally.[52] The cut-point at 13 or more indicates probable depression with a sensitivity of 86% and specificity of 78% in the postnatal period.[49] The optimal cut-off for probable major depression in the antenatal period may be higher (15 or more),[53], [54] thus we report multivariable results using this alternate cut-off. It should be noted that the EPDS is a screening tool that measures probable depression, and is not a clinical diagnosis of depression; however, to be succinct, we refer to an EPDS score ≥13 as antenatal or postpartum depression.

#### Child anthropometric measurements

Child length or height was assessed at birth, 6 months, 1 year, 2 years, and 3 years of age. Project Viva trained research staff completed measurements at birth, 6 months, and 3 years. We obtained measurements for one and two years of age from clinical records. In a previous study of patients in this group practice, Rifas-Shiman and colleagues found that clinical assessment of length or height in children under age 2 years systematically underestimated height compared to research standard methods, and they developed a regression equation to correct for this bias.[55] In the analyses presented here, we employed the corrected lengths for clinical measurements of children at 1 and 2 years. At age 3, staff also measured sitting height. We calculated leg length at age 3 as the difference between standing height and sitting height. Length/height was measured to the nearest 0.1 centimeter. Details regarding the age-three assessment have been published.[56] We calculated age- and sex-specific height-for-age z-score (HAZ) according to US national reference data for birth and ages six months, 1, 2, and 3 years.[57] We also calculated age- and sex-specific height-for-age z-score for age 3 years according to World Health Organization (WHO) Child Growth Standards[58], using the SAS program provided by WHO (available at http://www.who.int/childgrowth/software/en/, accessed 9/2/2010).

#### Sociodemographic factors and potential confounders

We chose variables that have been previously linked with perinatal depression or child height as covariates, see Table 1. Maternal age, race/ethnicity, household income, education, partnership status, height, pre-pregnancy BMI, and maternal report of the father's height were recorded at enrollment. We assessed maternal smoking during pregnancy by questionnaire and categorized responses into former smoker, never smoker, and smoked during pregnancy. We calculated pregnancy weight gain as pre-pregnancy weight subtracted from the last clinically recorded weight before delivery and categorized it according to the Institute of Medicine's gestational weight gain guidelines.[59] Presence of gestational diabetes or impaired glucose tolerance during pregnancy was collected from medical records. We identified women who used medication for depression during pregnancy by searching prescription data for the 90 days before the last menstrual period through delivery for 29 commonly used antidepressants. We calculated gestational age at delivery from the last menstrual period; if estimated gestational age based on last menstrual period differed by more than 10 days from the 2^nd^ trimester ultrasound estimate, we used the estimate from the ultrasound. We calculated birthweight for gestational age z-scores with use of US national reference data.[60]

**Table 1:** Characteristics of Project Viva Participants According to Maternal Antenatal and Postpartum Depression. Data from 872 mother-child pairs participating in Project Viva.

			Overall (n = 872)		Antenatal Depression				Postpartum Depression			
					No (n = 802)		Yes (n = 70)		No (n = 808)		Yes (n = 64)	
			mean/n	SD/%	mean/n	SD/%	mean/n	SD/%	mean/n	SD/%	mean/n	SD/%
Maternal characteristics												
	Age		33.0	4.5	33.0	4.4	32.1	5.0	33.1	4.4	31.6	5.4
	Race/ethnicity											
		Black	73	8.4	64	8.0	9	12.9	65	8.0	8	12.5
		Other	105	12.0	92	11.5	13	18.6	94	11.6	11	17.9
		White	694	79.6	646	80.6	48	68.6	649	80.3	45	70.3
	Education											
		Less than BA/BS	189	21.7	169	21.1	20	28.6	169	20.9	20	31.3
		BA/BS	348	39.9	322	40.2	26	37.1	327	40.5	21	32.8
		Graduate degree	335	38.4	311	38.8	24	34.3	312	38.6	23	35.9
	Married *		784	90.0	728	90.9	56	80.0	733	90.8	51	79.7
	Household income											
		< = $40,000	77	8.8	61	7.6	16	22.9	63	7.8	14	21.9
		40,001 to $70,000	195	22.4	176	22.0	19	27.1	176	21.8	19	26.7
		> = $70,001	600	68.8	565	70.5	35	50.0	569	70.4	31	48.4
	Pre-pregnancy BMI > = 25		288	33.0	259	32.3	29	41.4	260	32.2	28	43.8
	Height (inches)		65.1	2.7	65.1	2.7	64.6	2.4	65.1	2.7	64.8	2.9
	Paternal height (inches)		70.7	3.0	70.7	3.0	70.6	3.0	70.7	2.9	70.5	3.3
	BF duration (months) *		6.55	4.5	6.50	4.5	7.11	4.7	6.57	4.5	6.31	4.3
	Introduction of solid foods *											
		before 4 months	130	14.9	125	15.6	5	7.1	119	14.8	11	17.2
		4–5 months	623	71.5	566	70.7	57	81.4	578	71.6	45	70.3
		6 months or after	118	13.6	110	13.7	8	11.4	110	13.6	8	12.5
Infant characteristics												
	Girl		456	52.3	417	52.0	39	55.7	427	52.9	29	45.3
	GA at delivery (weeks)		39.6	1.7	39.6	1.7	39.5	1.5	39.6	1.7	39.5	1.6
	BW for GA (z value)		0.23	0.93	0.25	0.93	0.04	0.95	0.24	0.92	0.13	1.07
Child characteristics at 3 yrs												
	Age (months)		39.0	3.2	39.0	3.2	38.8	2.8	39.0	3.2	38.6	2.6
	Height (cm)		97.2	4.2	97.2	4.2	97.1	4.6	97.1	4.2	98.6	4.0
	HAZ		0.2	0.9	0.2	0.9	0.2	1.0	0.2	0.9	0.6	0.9
	Leg Length (cm)		41.6	2.6	41.6	2.5	42.0	2.8	41.6	2.6	42.4	2.3

BA/BS, Bachelor of Arts or Science; BMI, body mass index (kg/m2); BF, breastfeeding; GA, gestational age; BW, birth weight; HAZ, height-for-age z-score.

*Sample sizes are slightly different due to missing data: breastfeeding duration n = 869; marital status n = 871; introduction of solid foods n = 871.

#### Potential mediators

We considered the following to be potential mediators of antenatal and/or postpartum depression's impact on child height: subsequent depression (at 6 months and 1 year for antenatal depression and at 1 year for postpartum depression, each assessed by the EPDS), duration of breastfeeding, and age at which solid foods were introduced to the child, all of which were assessed by questionnaire. There was little overlap between depressive symptoms occurring in the perinatal period with depressive symptoms at 1 year postpartum: 18 subjects had elevated depressive symptoms at both 6 months and 1 year postpartum and 11 at both antenatal and 1 year postpartum. Additionally, we considered gestational age at birth and birthweight for gestational age potential mediators of the influence of antenatal depression on child stature.

### Statistical analysis

We examined two sets of outcomes: HAZ and leg length at age 3 years, and change in HAZ from birth through age 3. In both cases, we separately modeled the impact of antenatal (EPDS> = 13 and EPDS> = 15) and postpartum depression. For HAZ and leg length at age 3, we used multivariable linear regression models. In the base model (Model 1) for each exposure, we adjusted for child sex and age in months at assessment. In Model 2, we adjusted for sociodemographic and physiological factors related to maternal depression and/or child height. In Model 3, we additionally adjusted for gestational age at birth and birthweight for gestational age z-score; these variables may be mediators of the association of antenatal depression with height as well as confounders of the association of postpartum depression with height. To increase precision of the estimate of interest, if removal of a covariate did not substantially alter the effect estimate of the primary exposure, that covariate was not included in the models presented here. Covariates tested and not included were maternal marital status, education, smoking during pregnancy, gestational diabetes or impaired glucose tolerance during pregnancy, paternal height, and anti-depressant use during pregnancy. Our primary outcomes were HAZ according to CDC reference standards and leg length; we also ran our models using HAZ according to WHO reference standards because the CDC and WHO standards are based on different reference populations. If results are consistent using both standards, we have some assurance that estimated associations are not due to the choice of reference population.

Seventy mothers experienced depression during pregnancy and 64 at 6 months postpartum. Only 19 mothers experienced depression at both time points. Due to the relative lack of overlap in these exposures, we examined antenatal and postpartum depression separately. Estimated effects for antenatal and postpartum depression were similar whether we included these 19 subjects or not; thus they are included in both exposure categories.

Research-quality measurements of birth length were available in a subset of the study population (n = 516). In this subset, we found that controlling for birth length z-score in place of birthweight for gestational age z-score (available for the whole cohort) did not affect results, so we present results for the larger cohort only, and use birthweight for gestational age z-score as a control variable. Also in this subset of the study population, we conducted a direct test of reverse causation for the association between postpartum depression and height outcomes at age 3: we examined whether length at birth predicted postpartum depression, controlling for maternal age, race/ethnicity, height, pregnancy weight gain, household income, and child gender.

Potential mediators of the effect of antenatal depression were gestational age at birth, birthweight for gestational age,[46] breastfeeding duration, age of introduction of solid foods, and maternal depression at 6 months and 1 year postpartum. Potential mediators of the association of postpartum depression with height were breastfeeding duration, age of introduction of solid foods, and depression at 1 year postpartum. To test for mediation, we added potential mediators to the fully-adjusted models and assessed the magnitude of attenuation of the estimated effect of exposure to maternal depression.

For the outcome change in HAZ from birth to 3 years, we used longitudinal models allowing a random intercept, random slope, and an unstructured covariance matrix. Covariates were based on the fully-adjusted models presented in Tables 2 and 3. In models with antenatal depression as primary predictor, we modeled the change in HAZ from birth through age 3. In models with postpartum depression as primary predictor, we modeled the change in HAZ from age 6 months to 3 years. We entered time as a categorical variable, with each outcome assessment (birth, 6 months, 1, 2, and 3 years) as a category and tested for an interaction between maternal depression and time. All analyses employed SAS version 9.1 (SAS Institute, Cary, NC), proc glm for age 3 outcomes and proc mixed for longitudinal analyses.

**Table 2:** Difference in 3-year height outcomes among children exposed versus not exposed to antenatal depression, using 2 alternate cut-off values for the Edinburgh Postnatal Depression Scale (EPDS).

	EPDS> = 13		EPDS> = 15	
	Estimate (95% confidence interval)		Estimate (95% confidence interval)	
	Height-for-age z-score	Leg Length (cm)	Height-for-age z-score	Leg Length (cm)
Model 1	0.002 (−0.22, 0.23)	0.48 (−0.08, 1.04)	0.07 (−0.21, 0.36)	0.63 (−0.08, 1.34)
Model 2	0.01 (−0.20, 0.22)	0.45 (−0.07, 0.97)	0.13 (−0.14, 0.39)	0.74 (0.07, 1.40)
Model 3	0.03 (−0.17, 0.24)	0.48 (−0.03, 1.00)	0.16 (−0.10, 0.42)	0.79 (0.14, 1.44)
Model 4	−0.04 (−0.25, 0.17)	0.32 (−0.20, 0.84)	0.06 (−0.21, 0.32)	0.58 (−0.09, 1.24)
Model 5	−0.02 (−0.23, 0.19)	0.36 (−0.16, 0.89)	0.10 (−0.17, 0.37)	0.68 (0.01, 1.35)

Model 1 covariates: child sex and age at 3-year assessment.

Model 2: Model 1+ maternal: age, race/ethnicity, household income, height, pregnancy weight gain.

Model 3: Model 2+ gestational age at birth and birthweight for gestational age z-value.

Model 4: Model 3+ postpartum depression.

Model 5: Model 4+ breastfeeding duration and age of introduction of solid foods (n = 869).

Data from 872 mother-child pairs participating in Project Viva.

**Table 3:** Difference in 3-year height outcomes among children exposed versus not exposed to postpartum depression.

	Estimate (95% confidence interval)	
	Height-for-age z-score	Leg Length (cm)
Model 1	0.39 (0.16, 0.62)	0.97 (0.39, 1.56)
Model 2	0.35 (0.13, 0.57)	0.85 (0.31, 1.39)
Model 3	0.37 (0.16, 0.58)	0.88 (0.35, 1.41)
Model 4	0.37 (0.16, 0.58)	0.89 (0.36, 1.41)

Model 1 covariates: child sex and age at 3-year assessment.

Model 2: Model 1+ maternal: age, race/ethnicity, household income, height, pregnancy weight gain.

Model 3: Model 2+ gestational age at birth and birthweight for gestational age z-value.

Model 4: Model 3+ breastfeeding duration and age of introduction of solid foods (n = 869).

Data from 872 mother-child pairs participating in Project Viva.

## Results

Mothers in this sample were predominantly white, married, and had an annual household income over $70,000 (Table 1). Seventy (8.0%) participants experienced antenatal depression and 64 (7.3%) experienced postpartum depression. Women with antenatal depression were less likely to be married and more likely to have lower income, inadequate weight gain during pregnancy, and gestational diabetes or impaired glucose tolerance. Women with postpartum depression were younger, less likely to be married, had lower income, and their children were taller and had longer leg length at age 3.

In multivariable models predicting age 3 outcomes, there was some indication that exposure to antenatal depression was associated with greater leg length but not HAZ (Table 2). In a model controlling for potential confounders (Model 2), antenatal depression (EPDS> = 13) was associated with (0.45 (−0.07, 0.97)) greater leg length. Use of the more stringent definition of antenatal depression (EPDS> = 15) revealed a stronger association between antenatal depression and leg length in children (0.74 (0.07, 1.40)). Further control for depression at 6 months and 1 year postpartum did not materially alter results.

Postpartum depression was associated with greater HAZ and leg length. As Table 3 displays, after controlling for potential confounders (Model 3), compared to children not exposed to postpartum depression, exposed children had HAZ 0.37 (0.16, 0.58) and leg length 0.88 cm (0.35, 1.41) larger. Maternal height, birthweight for gestational age, non-white race/ethnicity, and lower household income were other factors associated with taller height in children (Table 4). The finding that black children of this age are taller is consistent with findings from a nationally-representative sample of young children.[61] Restriction to individuals without antenatal depression did not materially alter results, nor did further control for maternal depression at 1 year postpartum. There was no evidence for mediation by breastfeeding duration or age of introduction of solid foods, though longer duration of breastfeeding was an independent predictor of reduced HAZ and leg length. Each month of longer duration of breastfeeding was associated with a −0.02 (−0.03, −0.01) difference in HAZ and a −0.05 cm (−0.08, −0.01) difference in leg length. Additionally, there was no evidence of reverse causation: birth length was not a significant predictor of postpartum depression (OR = 1.39, 95% CI: 0.85, 2.27) in a logistic regression model controlling for maternal age, race, height, pregnancy weight gain, household income, and child gender.

**Table 4:** Factors associated with height outcomes at age 3 years.

		Estimate (95% confidence interval)	
Variable		Height-for-age z-score	Leg Length (cm)
Postpartum depression		0.37 (0.16, 0.58)	0.88 (0.35, 1.41)
Maternal race/ethnicity			
	Black	0.39 (0.19, 0.60)	2.04 (1.53, 2.56)
	Other	0.18 (0.00, 0.35)	0.57 (0.14, 1.01)
	White	0 (ref)	0 (ref)
Household income			
	< = $40,000	0.20 (0.00, 0.41)	0.33 (−0.19, 0.85)
	40,001 to $70,000	0.12 (−0.02, 0.25)	0.16 (−0.17, 0.50)
	> = $70,001	0 (ref)	0 (ref)
Maternal height (inches)		0.11 (0.09, 0.13)	0.24 (0.19, 0.29)
Birthweight for gestational age z-score		0.23 (0.17, 0.29)	0.35 (0.20, 0.51)

Multivariable linear regression model adjusted for maternal age and pregnancy weight gain and child sex, gestational age at birth, and age at outcome assessment as well as all variable in the Table.

Use of WHO reference standards revealed similar results as those displayed in Table 2 and Table 3. The estimated association between antenatal depression and WHO height-for-age z-score was −0.01 (−0.27, 0.24) in a model controlling for maternal age, race/ethnicity, household income, height, and pre-pregnancy weight gain (the same covariates as in Model 2 of Table 2). The estimated association between postpartum depression and WHO HAZ was 0.28 (0.02, 0.54) controlling for the same covariates as in Model 3 of Table 3.

**Figure 1:**
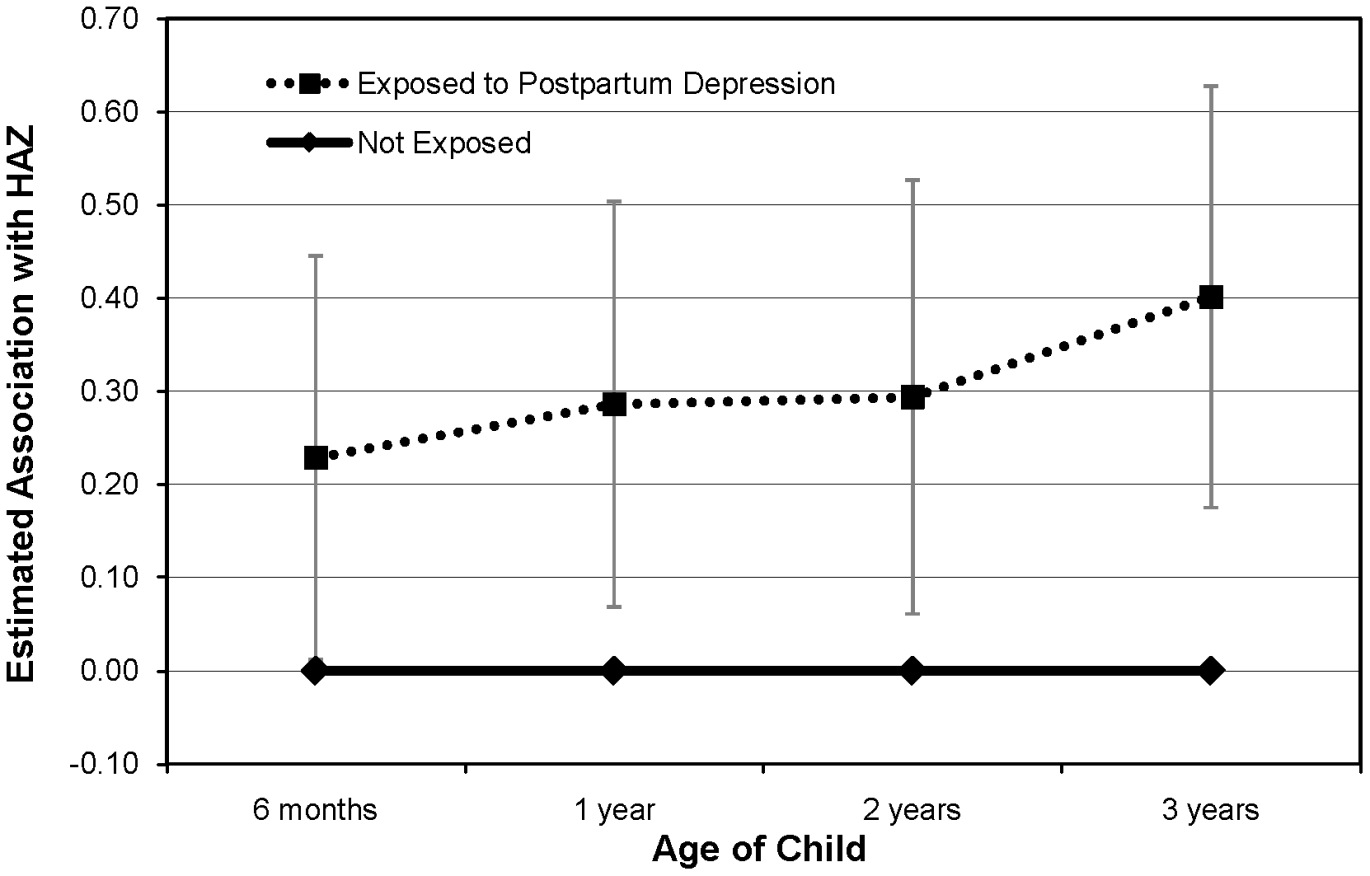
Estimated longitudinal association of postpartum depression with child height-for-age z-score (HAZ). Data from 872 mother-infant pairs participating in Project Viva. Multivariable regression estimates adjusted for maternal: age, race/ethnicity, household income, height, and weight gain during pregnancy and child: sex, gestational age, and birthweight for gestational age z-score. Bars indicate 95% confidence intervals. Exposed to postpartum depression (dotted line), not exposed to postpartum depression (solid line).

In longitudinal models, the association between postpartum depression and child HAZ from 6 months through age 3 was similar for each age (Figure 1), thus we did not include a postpartum depression by age interaction term in the final model. The association between postpartum depression and child HAZ was 0.29 (0.11, 0.47) at each time point. Similar to age 3 findings, antenatal depression was not associated with HAZ from birth through age 3: the estimated association over all time points was −0.01 (−0.19, 0.17).

## Discussion

This study did not find evidence for the hypothesis that maternal depression is associated with reduced height in children. Contrary to expectations, maternal postpartum depression was associated with greater child total height and leg length in this relatively privileged sample in a high income country. Postpartum depression was associated with 0.37 higher HAZ, which translates into an approximately 1.5 centimeter difference in 3-year-olds of average height (94.5 cm) in the US.[57] This finding was independent of potential confounders, including maternal sociodemographic factors and height as well as child size at birth. We found no evidence for mediation by duration of breastfeeding or age of introduction of solid foods, nor any evidence for reverse causation. Additional control for depression at 1 year postpartum did not diminish the estimated effect for postpartum depression at 6 months, nor was there a main effect of depressive symptoms at 1 year postpartum on height outcomes, suggesting that exposure to maternal depression earlier in life is more important.

Antenatal depression when defined by the more stringent criteria of EPDS> = 15 was associated with greater leg length in children. Comparing results using alternate cut-off values for the EPDS suggests a dose-response relation between antenatal depressive symptoms and linear growth outcomes in children.

Our results contrast with several studies in developing countries that report either a cross-sectional or prospective association between maternal depressive symptoms or clinical depression and reduced child height or increased risk of stunting.[39]–[42] However, not all studies in developing countries support an association between maternal mental health and reduced height in children.[43]–[45] It should also be noted that maternal depression has been found to be associated with both reduced weight[32], [34], [39], [62] (for a review, see Stewart, 2007 [38]) and *increased* weight[63] in different settings; thus, there is prior evidence that maternal depression may have different effects in different contexts.

It is unclear what mechanisms may explain divergent findings between developing countries and the US. One possible source of difference is risk of nutritional deficiency and serious childhood illness across these settings. In resource-poor settings, maternal depression may be associated with or lead to lack of sufficient food for the household or inadequate care of childhood illness, both of which may contribute to shorter stature in children. In Project Viva, a middle and upper class sample in the US, there are likely very few families who cannot obtain adequate food, though we do not have data to examine this issue directly. Similarly, severe childhood illness that may affect height is limited in the Project Viva sample. Other notable differences between the study population examined here and studies in low and middle income countries include high levels of maternal education, relatively low levels of maternal depression, and low rates of child stunting. We may speculate that in the rather privileged families enrolled in Project Viva, one threat (maternal depressive symptoms) is not enough to interrupt children's growth, while in developing countries in the context of several threats (including food insecurity, low education, and high rates of stunting) and fewer resources, maternal depression is an additional burden that influences children’s growth.

Another possible reason for the different findings is that many of the studies in developing countries used a dichotomous outcome of stunting, while we used height as a continuous outcome. We cannot examine whether this may help explain divergent results because only 7 subjects in our sample met criteria for stunting (HAZ<−2). Reports from Brazil, however, support the notion that maternal depression is associated with stunting,[41] but not height when examined as a linear outcome variable.[44] Future research may endeavor to examine these possible explanations further.

Interpretation of our results should be in light of the study's limitations. Mother-child pairs included in our analysis represent 37% of enrolled women. It is possible that this loss to follow-up introduced some bias, though it is unlikely that it induced a positive association between postpartum depression and child height. This loss to follow-up may limit the generalizability of our findings. The mothers included are generally well-educated, of middle to upper income, and all have health insurance. It is possible that the association between maternal depression and child height may be different in more at-risk populations. Similarly, though we statistically control for important variables related to maternal depression and height, residual confounding is always a concern. However, while residual confounding could have resulted in over- or under-estimates in our study, it is unlikely that the direction of effect would have changed with better control of confounding. Regarding our exposures, we rely on self-report of depressive symptoms, not clinically-defined depression, and depressive symptoms were assessed at only two time points in the perinatal period, which likely resulted in misclassification of exposure. This may be particularly important for postpartum depression: we assessed postpartum depression at 6 months, but the prevalence of postpartum depression is highest in the 2^nd^ and 3^rd^ months postpartum.[21] Misclassification of our exposure is likely nondifferential with respect to the outcomes, which would attenuate results towards the null. Finally, due to the relative independence of antenatal and postpartum depression, our results generalize primarily to women who have either antenatal or postpartum depression, although inclusion of women with both did not materially alter the estimated effects in this cohort of women. Given these limitations as well as the unexpected findings, replication of these results in another US-based cohort is particularly important.

In considering the mechanisms underlying the current findings, although many factors influence height, very few are likely to mediate an effect of postpartum depression on linear growth. One possible pathway is suboptimal nutritional intake among children of depressed mothers. Depressed mothers may have difficulty recognizing signs of satiety in their children, use more mechanistic methods of feeding, or use restrictive feeding practices.[64], [65] Secondarily, maternal depression negatively affects mother-child interactions,[24], [25] which may have implications for the child's nutritional intake. Depressed mothers may have more unhealthful behaviors, such as dysregulated eating behaviors (including over-eating and eating high-calorie foods) and limited physical activity, and their children may have similar behavior patterns. It is possible that these behaviors lead to increased energy intake in infants; in a related analysis of Project Viva participants, maternal postpartum depression was associated with greater overall adiposity in children at age 3 years.[66] We may speculate that increased caloric intake could have contributed to the greater linear growth observed here through actions of growth hormone or insulin-like growth factors; however, this study did not measure caloric intake or growth hormone, thus we were not able to test these possible mechanisms. That children exposed to postpartum depression showed greater height as early as 6 months of age suggest that influences before this age are likely to be at play.

Another possible pathway is through cortisol. In two studies, postpartum depression was associated with increased cortisol and cortisol variability in newborns and adolescents.[27], [67] Postpartum depression, through its adverse effects on parenting behavior and the social environment, can produce more stressful experiences for the child. Stressful experiences, in turn, lead to elevated cortisol levels and increased cortisol reactivity in children.[68], [69] Cortisol has a complex relationship with growth hormone: while chronic hypercortisolism leads to reduced growth hormone and reduced growth, small increases in cortisol stimulate growth hormone secretion and production.[70] It is possible that increased cortisol in the children exposed to postpartum depression had a stimulating effect on growth hormone and thereby increased height. Unfortunately, we did not measure these hormones in the current study.

The current study has several strengths. Project Viva is a prospective study with repeated outcome measurements, which allowed us to establish temporal order between exposure and outcomes as well as examine change over time in outcomes. This study assessed depressive symptoms both during pregnancy and during the postpartum period, allowing us to assess the contribution of each exposure individually. In addition, the primary outcomes of this study were collected using research-standard protocols, improving the precision with which we estimate relations.

Our study did not support the hypothesis that maternal depression is associated with shorter stature in children. To our knowledge, this study is the first to report an association between maternal depression and increased height in children. As one of the first studies to examine these relationships in a US sample, these findings should be replicated in other, more diverse samples in the US and other developed nations. Our findings suggest that postpartum depression has important influences on behavior or physiology early in life that translate into different growth patterns in children. Public health implications of our findings await elucidation of the contexts in which and mechanisms through which maternal depression may affect growth in children. Future research should study possible mechanisms that may account for the different linear growth seen in children of depressed mothers, including mother-infant interactions, dysregulated feeding and/or eating behavior, as well as underlying hormonal differences.
